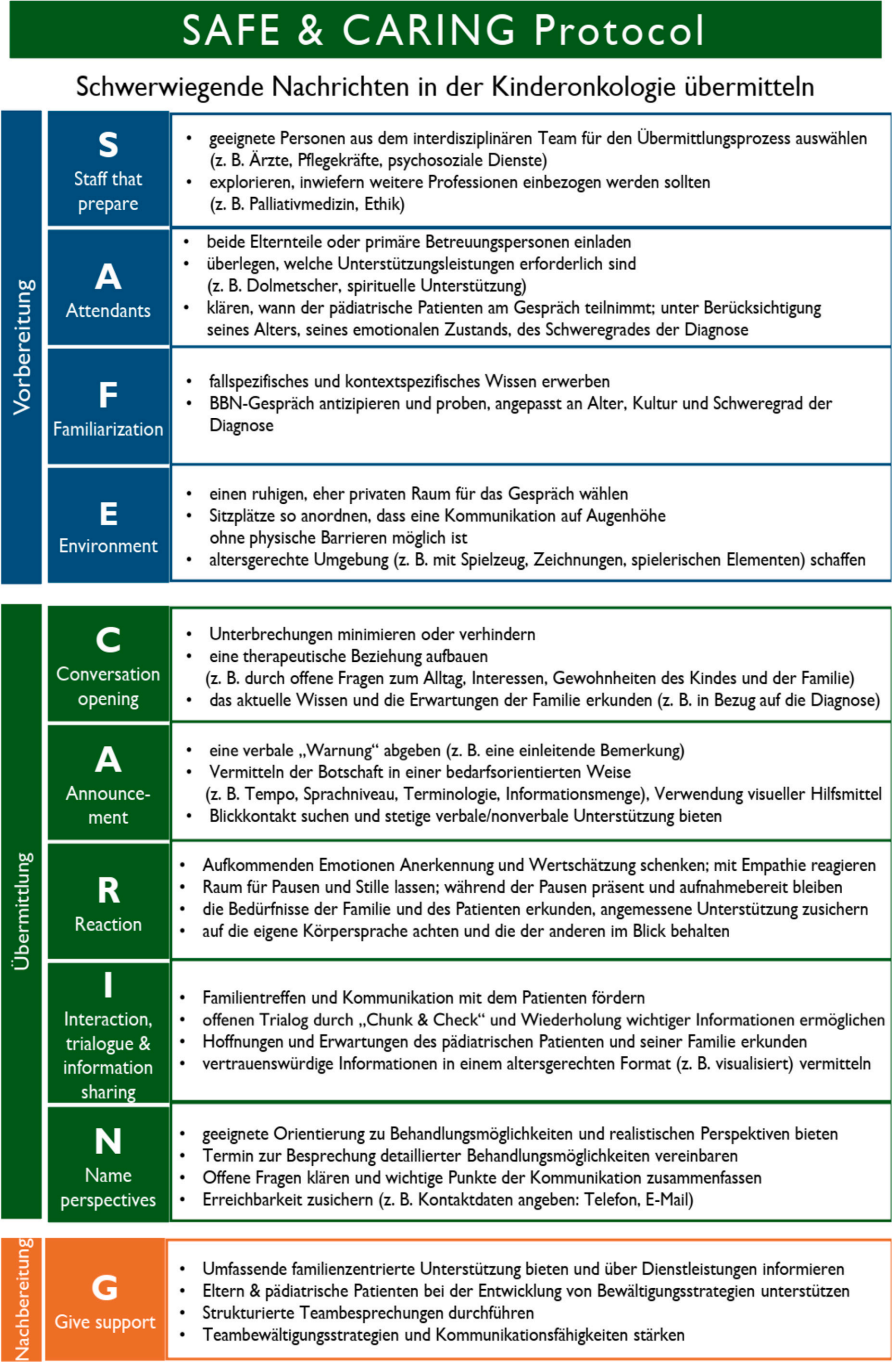# Mapping review on breaking bad news and design of SAFE & CARING protocol for pediatric hematology-oncology

**DOI:** 10.1016/j.pecinn.2026.100464

**Published:** 2026-03-02

**Authors:** Theresia Krieger, Lisa Frey, Marc Hoemberg, Kerstin Dittmer

**Affiliations:** a Medical Psychology | Neuropsychology and Gender Studies & Centre for Neuropsychological Diagnostics and Intervention (CeNDI), Faculty of Medicine and University Hospital Cologne, University of Cologne, Germany; b Medical Faculty, University of Cologne, Cologne, Germany; c Department of Pediatric Oncology and Hematology, Children's Hospital, University of Cologne, Cologne, Germany

**Keywords:** Pediatric hematology-oncology, Breaking bad news, Protocol, Mapping review, Co-creative design

## Abstract

**Objective:**

(1) to map existing Breaking Bad News (BBN) protocols and (2) to develop a context-specific, practical “first aid” triadic communication protocol, for the German pediatric hematology-oncology (PHO) setting where no such tool currently exists.

**Methods:**

A mapping review was conducted to systematically identify existing BBN protocols. Building on this, theoretical and practical evidence was integrated in a collaborative concept mapping exercise with German PHO experts, resulting in the extraction of PHO-specific components, develop BBN categories, refine key concepts, and the design of a new context-specific protocol.”

**Results & innovation:**

Of the 28 reviewed protocols, 20 contained potential PHO-relevant content and were analyzed by time, origin, and target population. Seventeen protocols included components suitable for PHO. Integrating these components with practical evidence led to the creation of the acronym-based ‘SAFE & CARING' BBN protocol. It covers all three BBN phases: ‘SAFE’ supports preparation, while ‘CARING' guides delivery and follow-up. It conceptualizes BBN as interdisciplinary task and explicitly addresses the requirements of triadic communication.

**Conclusion & innovations:**

The ‘SAFE & CARING' protocol is a structured, hands-on first-aid tool for interdisciplinary PHO teams, particularly in settings with limited formal training in triadic communication. Its key innovation includes the collaborative development process and the integration of triadic communication as an overarching principle.

## 1 Introduction

### 1.1 Defining BBN

Breaking Bad News (BBN) presents a major challenge for clinical practice in pediatric hematology-oncology (PHO) [1], [2], [3]. BBN is defined as providing any information which adversely and seriously affects an individual's view of his or her future [4]. It may entail the transmission of information about the child's cancer diagnosis, the potential for recurrence or progression of the disease or the availability of limited treatment options [5]. Although BBN is the established term, it is increasingly debated, with growing awareness that ‘sharing serious information’ may be preferable for conceptual and psychological reasons [6].

### 1.2 Communicating BBN in PHO

Six principles of communication are considered as crucial when delivering serious news: exchanging information, responding to emotions, managing uncertainty, fostering healing relationships, making decisions and enabling patient-self management [7]. BBN is conceptualized as a circular process [8], which is not confined to a single moment but instead comprises three distinct phases: preparation, delivery, and follow-up, including adjusting and coping [9].

In PHO, BBN is a complex triadic process involving the healthcare professional, the child, and the family [3], [10], [11], [12]. Unlike the typically dyadic communication in adult oncology, HCPs must balance the informational and emotional needs of both patient and parents.

The extent to which communication unfolds in a dyadic or genuinely triadic manner may vary depending on the child's developmental stage and communicative capacity, as well as the family's cultural or religious background [14], [15], [16]. The involvement of children in clinical encounters poses distinct communicative challenges, including age-appropriate information delivery and the management of emotional dynamics within the family context [13], [14].

Parental emotional burden, often characterized by shock and distress, may further dominate interactions and influence communicative dynamics [15], [16][Although widely acknowledged, triadic communication remains underdeveloped in practice and is often perceived as a ‘black box’, with little guidance on reconciling differing perspectives [1], [3], [13].

In this manuscript, we use the term *trialogue* to describe BBN in pediatric PHO, with healthcare professionals delivering the message and both parents and pediatric patients as the recipients.

### 1.3 Challenges and consequences

The quality of BBN is considered a critical factor influencing the patient's treatment journey, shaping adherence, coping with treatment challenges, and cancer-related self-efficacy in managing emotional distress [16], [17]. Limited resources, time constraints, lack of guidance, and insufficient BBN communication skills may negatively affect pediatric patients, their families, and HCPs [5], [18], [19], [20]. Beyond the serious content of the message itself, the recipients' (pediatric patients and their families) responses are shaped by multiple factors, including personality, social support, religion, culture, and current psychological state [21], [22].

BBN is often perceived as primarily the physician's responsibility, a role that is frequently associated with high levels of stress. However, other HCPs also report significant emotional burdens, including anxiety, feelings of failure, guilt, exhaustion, and frustration [23], [24]. Moreover, studies show that many physicians exhibit measurable physiological stress reactions during BBN situations [18].

Internationally, many HCPs report lacking sufficient communication skills for BBN. In a survey conducted across 40 countries, only one-third of respondents—including physicians, nurses, and students—indicated that they had received formal communication training [25]. Where training is available, it is often described as informal, locally organized, and lacking structured curricula [23]. Consequently, many HCPs feel unprepared, particularly when tasked with delivering serious or life-altering news to patients or their families [26]. For example, in a United States-based survey, only 64% of pediatric chief residents felt adequately prepared to deliver bad news to a parent, and just 31% felt prepared to do so with a child [27]*.* To date, no data are available regarding the extent or nature of BBN training among HCPs in Germany, nor whether existing training programs are tailored to the specific demands of the German PHO context.

### 1.4 Addressing BBN-need with a communication strategy

BBN in PHO is a complex and particularly sensitive challenge. Its quality has a significant impact on pediatric patients, their families, and HCPs. It requires specific knowledge, skills, and attention. To improve the quality and consistency of BBN, a context-specific, comprehensive communication strategy appears essential. Such a strategy should ideally include three components: (1) structured communication skills training; (2) a comprehensive communication guide (like a scientific text book); and (3) practical communication protocol that can serve as a “first-aid” tool or standard operating procedure, particularly in situations where formal training or access to detailed guidelines is limited.

In Germany, such an integrated communication strategy is currently lacking. To date, only one component—a comprehensive communication guide—has been developed [28] while structured training and protocol-based tools are largely absent.

#### 1.4.1 Communication skills training

Internationally, the availability of general communication skills training (CST) in PHO remains limited, the body of evidence is heterogeneous, and the absence of standardized outcome metrics is a matter of concern [29]. Nevertheless, several training programs have been developed over the last decade. In the United States, Peds OncoTalk was adapted from VitalTalk [30], and the Canadian Serious Illness Communication Program (SICP) is currently in operation [31]. The Diplôme Inter-Universitaire d'Oncologie Pédiatrique (DIUOP) is the only European program in pediatric oncology, offered in French for physicians involved in the care of children with malignancies [32]. In Germany, SICKO (Security training in PHO) addresses the topic communication in a 1.5-h session, but not with focus on BBN [33]. Notably, no BBN-specific communication training has yet been implemented in Germany, highlighting a significant gap.

#### 1.4.2 Communication guide

Internationally, comprehensive communication guides in PHO offer practice-oriented, textbook-style or web-based resources for delivering serious news. These guides integrate evidence-based recommendations, case examples, and insights from patients, families, and HCPs [34], [35], [36]. They are commonly used for self-directed learning and formal communication training, emphasizing the value of structured, context-sensitive guidance.

In Germany, the project *Orientierungskompass zur Übermittlung schwerwiegender Nachrichten in der Kinderonkologie* (OKRA) led to the development and implementation of a national communication guide for PHO, known as the “OKRA Compass” (11/2023–12/2024) [37], [38], [39]. This guide is based on practical experience and incorporates perspectives from HCPs, patients, families, outpatient support services, and researchers [38]. It offers practice-based recommendations for preparing, delivering, and following up on BBN situations in PHO, and includes visual aids, prompts, case scenarios, and reference materials to support everyday practice.

#### 1.4.3 BBN protocols

Existing BBN protocols - such as SPIKES (Setting, Perception, Invitation, Knowledge, Emotion, Strategy/Summary) and BREAKS (Background, Rapport, Explore, Announce, Kindle, Summarize) - were developed largely based on theoretical frameworks or expert opinion, without involving pediatric patients or their families [19], [40], [41]. They primarily address adult oncology contexts and do not consider the specific demands of triadic communication in pediatric settings, nor the structural characteristics of national healthcare systems. While BBN is a routine yet emotionally challenging task in PHO, clear and systematically developed, evidence-based protocols to support HCPs in structuring this process remain scarce [13], [42], [43], [44], [45]. In Germany, no PHO-specific BBN protocol has been developed or systematically implemented to date.

In this study, the term *protocol* refers to a structured, logical, and easy-to-apply “first-aid” communication tool designed to guide HCPs through the phases of BBN with patients and families. Unlike comprehensive guides, a protocol serves as a practical, immediately accessible “handrail” - especially in situations where formal training was not attended or the reading of broader guidelines is not possible, e.g., due to lack of time. The protocol focuses on the communication process, not the clinical content, and is not intended to replace training but to supplement it in acute settings.

### 1.5 Objective

We aim to (1) map existing BBN protocols and (2) develop a context-specific protocol for the German PHO, supporting interprofessional, triadic communication.

Our protocol is specifically intended to support the systemic, interdisciplinary nature of BBN in PHO. Since delivering serious news often involves collaboration among physicians, nurses, psychosocial professionals, and other team members, the protocol aims to facilitate this team-based, triadic communication process, addressing a gap not covered by existing adult-focused or single-profession tools.

## 2 Methods

### 2.1 Study design and execution

To obtain a comprehensive and visual overview, we conducted a mapping review, a form of a scoping review. This approach is transparent, rigorous, and systematic, allowing the categorization and visualization of heterogeneous evidence to identify gaps and provide a structured understanding of the research area [46]. It aligns with the exploratory aims of our study and facilitates the systematic collection and presentation of existing BBN protocols, as well as the development of a new context-specific protocol for the PHO [47], [48].

**Fig. 1:**
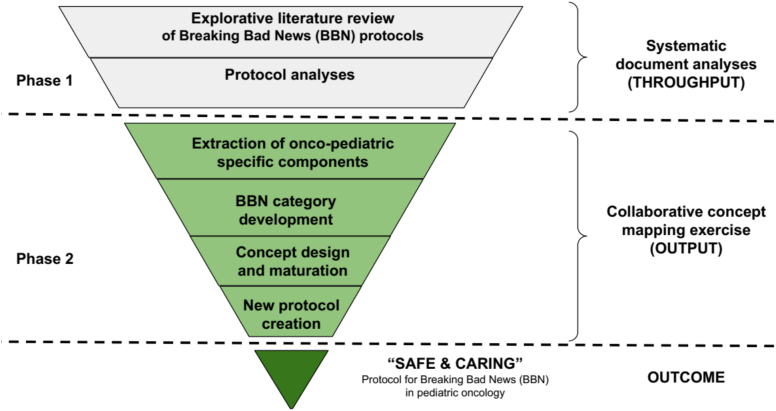
A qualitative mixed methods mapping review depicting the process from systematic document analysis to creating the outcome “SAFE & CARING” protocol for BBN in pediatric hematology-oncology (study design).

Our explorative study comprised two study phases and six consecutive working steps (Fig. 1). The final outcome was a new protocol, “SAFE & CARING,” designed explicitly for PHO (Fig. 5).

A qualitative mixed-methods design was employed, combining two qualitative data collection methods [49], [50]. In Phase 1, systematic document analyses were conducted to generate evidence-based knowledge on existing BBN protocols using a top-down approach [51], resulting in a context-specific overview (throughput). In Phase 2, the evidence from Phase 1 was critically reflected upon and integrated with current practice-based, context-specific insights through a collaborative concept mapping exercise [52]. This process, considered as the output, led to the systematic development of a new PHO-specific protocol (outcome) (see Fig. 1).

This study was conducted by four individuals with expertise in healthcare development, health system research, medicine, nursing sciences, and public health. Systematic document analysis was conducted by FL (Phase 1); all authors contributed to the collaborative concept mapping process (Phase 2).

#### 2.1.1 Phase 1: systematic document analyses

**Fig. 2:**
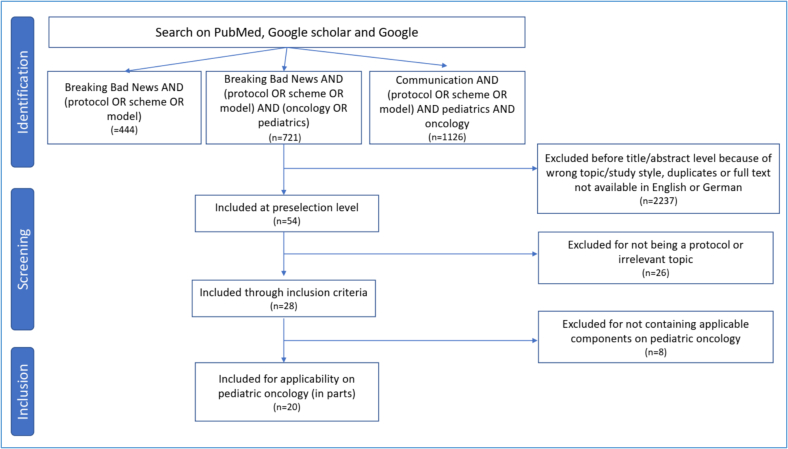
Adapted PRISMA-ScR flowchart depicting the data selection process in Phase 1.

An exploratory, mapping review was conducted to comprehensively identify and categorize existing BBN protocols, following the PRISMA-ScR guidelines (Checklist, see Supplement 1) [53]. This approach—considered a subtype of scoping review—is particularly suited for broad and heterogeneous evidence landscapes and allows for structured visual presentation of findings (Fig. 2).

##### 2.1.1.1 Step 1: explorative literature review

In order to identify the earliest protocol, the timeframe for publication dates was not narrowed. A structured literature search was conducted in between March and April 2024. The following three Boolean search strings were used in both databanks PubMed and Google Scholar: (1) “breaking bad news” AND (protocol OR scheme OR model), (2) “breaking bad news” AND (protocol OR scheme OR model) AND (oncology OR pediatrics), and (3) “communication” AND (protocol OR scheme OR model) AND pediatrics AND oncology. Given the considerable number of search results returned by Google Scholar, the results were sorted in descending order of relevance. The screening process was concluded once 20 or more consecutive search results were identified as irrelevant to achieving this study's objectives.

The inclusion and exclusion criteria were predefined and consistently applied throughout the screening process. The initial search also outlined as phase 1 and screening of articles were conducted by a single reviewer (FL). While this approach may limit the potential for independent verification, careful adherence to the established criteria was maintained to ensure consistent and objective selection.

The inclusion criteria were as follows: the full text must be available in English or German, and the article must contain a protocol designed for BBN. Articles that mention protocols without providing details (e.g., those that aim to validate a protocol in clinical use) were excluded. Protocols that lacked a clear connection to their use in a hospital setting (e.g., for marketing or product management) or lacked components transferable to a pediatric context were also excluded (e.g., for diagnostic non-disclosure due to cultural dilemma, oral medicine, neonatology or focus on video call only).

##### 2.1.1.2 Step 2: protocol analyses

**Fig. 3:**
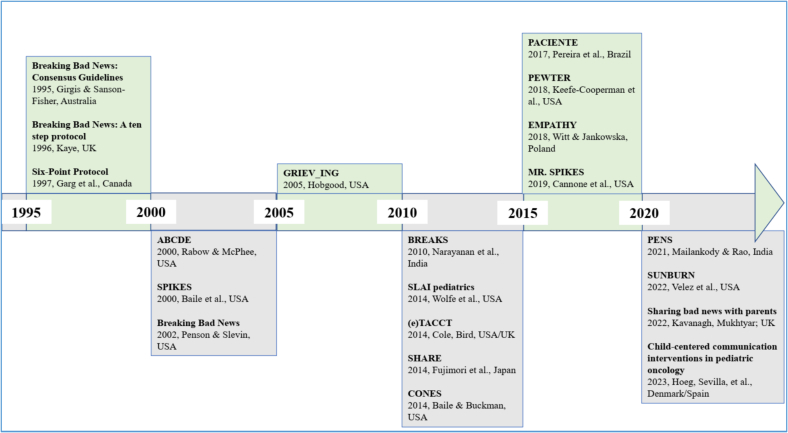
Overview of the BBN protocols that include issues that are transferable to pediatric hematology-oncology (Map 1).

All oncology-related protocols were critically analyzed. First, a comprehensive overview of all existing BBN protocols was compiled, including details such as their intended setting, geographic context, protocol name, and authors (Annex 1). Next, only those protocols containing elements potentially useful for BBN in the onco-pediatric context were examined in detail (Fig. 3). Protocols not meeting this criterion were excluded from further consideration.

#### 2.1.2 Phase 2: collaborative concept mapping exercise

In order to develop a context-specific protocol for German PHO, supporting interprofessional, triadic communication in a setting where no such tool currently exists (objective 2 of this study), a collaborative concept mapping exercise was conducted (Fig. 1; Phase 2, steps 3–6) [52]. This structured method is helpful for visually organizing and connecting key concepts and is often used to integrate diverse forms of evidence and develop theory-informed frameworks [54].The iterative co-creative working process facilitated the integration of both theoretical and practical evidence.

##### 2.1.2.1 Step 3: extraction of onco-pediatric-specific components

The remaining protocols (Fig. 3) were systematically reviewed by the research team using a collaborative concept mapping approach. Each protocol was analyzed in detail to identify components, strategies, and principles that might be relevant for BBN in the PHO. Team members discussed and extracted elements they considered potentially useful based on their professional expertise and contextual knowledge. The protocols were organized chronologically to help trace when specific components first appeared (Fig. 3).

##### 2.1.2.2 Step 4: BBN category development

The extracted components were organized to reflect the typical progression of a BBN in the PHO.

All components were clustered in categories. Then the categories were labeled with a heading/name. Ultimately, each extracted component was then assigned to one of these categories in group discussion till consensus was achieved. Finally, the research team collaboratively classified the categories to one of the phases in the BBN process: “preparation”, “delivery transmission,” or “follow-up” (Table 1).

**Table 1:** The components allocated to the three consecutive BBN phases and 10 newly designed categories (Map 3).

Preparation phase
1. **Specialties that prepare** • Multidisciplinary team (Wolfe et al., 2014) • Support/care by nurses (Velez et al., 2022) • Involve professional who is used to dealing with children (Girgis & Sanson-Fisher, 1995)
2. **Attendants** • Both parents (Kavanagh & Mukhtyar, 2022) • Support person/appropriate family (Rabow & McPhee, 2000) • Patient participation (Wolfe et al., 2014)
3. **Familiarization** • Anticipate discussion (Girgis & Sanson-Fisher, 1995) • Anticipate practice (Rabow & McPhee, 2000) • Prepare expert knowledge (Narayanan et al., 2012)
4. **Environment** • Quiet/private place (Girgis & Sanson-Fisher, 1995) • Adequate seating (Rabow & McPhee, 2000) • Physical space comfortable for children/toys, show play (Høeg et al., 2023) • No barriers on eye level (Narayanan et al., 2012)

Transmission phase
5. **Conversation opening** • No interruptions (Girgis & Sanson-Fisher, 1995) • Open questions (Garg et al., 1997) • Ask about life and write down, follow up on it (Hoeg et al., 2023) • Assess understanding (Girgis & Sanson-Fisher, 1995) / level of denial (Garg et al., 1997) • Response will provide an appropriate starting point (Girgis & Sanson-Fisher, 1995) • Is more information wanted? (Kaye et al., 1996)
6. **Announcement** • Warning shot (Kaye, 1996) • Eye contact (Garg et al., 1997) • Provide information simply and honestly (Girgis & Sanson-Fisher, 1995) • Use same vocabulary (Garg et al., 1997) • Use visual aids (Wolfe et al., 2014), • Drawings (Cole & Bird, 2013) • Appropriate speed of conversation (Fujimori et al., 2014) • Avoid technical jargon/euphemisms (Girgis & Sanson-Fisher, 1995) • Avoid platitudes/false sympathy (Velez et al., 2022) • Avoid “nothing more can be done” (Girgis & Sanson-Fisher, 1995)
7. **Reaction** • Pause to process (Penson, Slevin, 2002) • Allow silence (Rabow, McPhee, 1999) • Encourage to express feelings freely, accept/validate feelings (Girgis, Samson-Fisher, 1995) • Provide tissues (Girgis, Samson-Fisher, 1995) • Reassure about pain, suffering, abandonment (Rabow, McPhee, 1999) • Body language (Garg et al., 1997) • Ask about how the child would like you/the parents to react (Hoeg et al., 2023) • Appropriate touch (Girgis, Samson-Fisher, 1995) • Empathic answer (Girgis, Samson-Fisher, 1995)
8. **Interactive trialogue and Information back up** • “chunk & check” (Garg et al., 1997) • Encourage discussion (Wolfe et al., 2014) • Combine empathetic/exploratory/validating statements (Baile et al., 2000) • Explore patient's expectations/hopes (Baile et al., 2000), no unrealistic hope (Narayanan et al., 2012), do not take away realistic hope (Witt & Jankowska, 2018) • Encourage family meetings (Girgis & Sanson-Fisher, 1995) • Written/diagram/pamphlet information (Girgis & Sanson-Fisher, 1995) • Links to reliable internet sites (Mailankody et al., 2020) • Repeat/revise as needed (Girgis & Sanson-Fisher, 1995) • Ask patient to repeat their understanding of news (Rabow & McPhee, 2000) • Support parents in interaction with their child (Høeg et al., 2023)
9. **Name perspectives** • Summarize (Garg et al., 1997) • Discuss treatment options (Girgis & Sanson-Fisher, 1995) • Address further needs (Rabow & McPhee, 2000) • Provide contact information (Girgis & Sanson-Fisher, 1995)

Follow-up phase
10. **Give support** • Provide information about support services (Girgis & Sanson-Fisher, 1995) • Debrief with team members (Wolfe et al., 2014) • Process your own feelings (Rabow & McPhee, 2000) • Be available (Girgis & Sanson-Fisher, 1995)

##### 2.1.2.3 Step 5: concept design and maturation

The emerging concept was refined through several iterative cycles involving team discussions and feedback. During this process, the team worked to identify appropriate and precise terminology to describe the content of the newly formed categories. Each component was critically reviewed in relation to real-world experiences and practices within the German PHO, ensuring that the labels and structure reflected both theoretical coherence and practical relevance.

##### 2.1.2.4 Step 6: new protocol creation

The finalized concept was translated into a structured protocol format. The research team generated synonyms for category headings to create a suitable acronym. Multiple options were considered before reaching consensus on one that best supported practical use. The acronym, serving as a mnemonic aid for recall and organization, emerged organically rather than guiding the design from the outset. Extracted components were mapped onto the three BBN phases—preparation, transmission, and follow-up—and aligned with the acronym to ensure clinical usability. Descriptions and activity prompts were refined to match PHO-specific terminology, communication practices, and workflow. The result is a context-sensitive, evidence-informed protocol to support HCPs in delivering bad news, with the acronym as an optional learning and application tool (Fig. 5).

## 3 Results

### 3.1 Mapping the theoretical evidence

#### 3.1.1 Step 1: explorative literature review

From the initially identified 2291 articles, 2237 were excluded (Fig. 2). Following a more comprehensive examination of the remaining preselected papers (*n* = 54), 26 were excluded due to the absence of protocols or irrelevance to the study topic based on their full text. Therefore, 28 BBN protocols were identified, which are presented chronographically (Annex 1). The first protocol emerged in Australia in 1995. Most protocols were designed in the USA (*n* = 9), followed by the UK (*n* = 4), India (*n* = 3), Brazil (*n* = 2), Poland (*n* = 2), Australia (*n* = 1), Canada (*n* = 1), Japan (*n* = 1), Iran (*n* = 1), Pakistan (*n* = 1), Ireland (*n* = 1), Germany (*n* = 1), and Denmark/Spain (*n* = 1).

#### 3.1.2 Step 2: analysis of oncology-specific protocols

The various components of the 28 BBN protocols (Annex 1) were analyzed and evaluated for their applicability to pediatric oncology. This process excluded eight protocols, leaving 20 that were included in Map 1 (Fig. 3), originating from the USA (*n* = 9), the UK (*n* = 3), India (*n* = 2), Australia (*n* = 1), Canada (*n* = 1), Poland (*n* = 1), Japan (*n* = 1), Brazil (*n* = 1), and Denmark/Spain (*n* = 1).

Of the 20 final BBN protocols deemed suitable for use in a pediatric context in parts, only two were initially intended to be used in a PHO (Fig. 3). Wolfe et al. [55] published the first protocol that was based on the SPIKES protocol [19], which was designed for use in adult oncology. Wolfe et al. [55] aimed to develop pediatric hospital guidelines with associated team training to enhance physician skills in BBN within a pediatric context. Thus, an interdisciplinary task force with expertise in pediatric HCPs and parents from pediatric patients added their accumulated knowledge and experience to the existing SPIKES protocol. Høeg et al. [56] published the second protocol, which was tailored to pediatrics and also toward oncology, based on a scoping review. They aimed to design and propose a child-centered communication tool based on the SPIKES protocol [19] and insights gained from the scoping review. The six core communication functions of the National Cancer Institute (healing relationships, information, responding to emotions, managing uncertainty, making decisions, and self-management) were used as a source of information [7]. Finally, the recommendations were categorized according to age group to accommodate the varying needs of different age groups.

The remaining 18 protocols address BBN, albeit not within the pediatrics context. In order to gain further insight into the selected protocols and ascertain the validation and utilization of the respective protocols in pediatrics, we have attempted to contact the authors. We were able to contact 12 authors using the most recent contact information available; however, attempts to contact the remaining eight authors were unsuccessful. Only five responses were received. Two protocols have been validated via self-assessment [55], [57]. One protocol is currently used in pediatrics [55]. Another is to be modified further and tested in a randomized control study [57]. One protocol is used in pediatrics, though it lacks validation [58].

Wolfe et al. [55] created a pediatric haemato-oncology teaching program based on their protocol. Parents from the respective departments are both part of the research group that created the protocol/program and part of the teaching team (e.g., as drama patients). Fujimori et al. [57] interviewed one young patient (AYA group) to develop their protocol. Their team included oncologists (not specified whether pediatric), mental health care providers, psychiatrists, psychologists, and a nursing staff member. They created a training program based on their protocol.

Further answers were received from the corresponding authors of Rabow & McPhee [42]; Pereira et al. [59] and Høeg et al. [56] negating both, validation and utilization in a pediatric context. The protocol of Pereira et al. [59] is planned to be validated and introduced to use in pediatrics.

### 3.2 Designing a new pediatric-specific BBN protocol

#### 3.2.1 Step 3: extraction of context-specific components

**Fig. 4:**
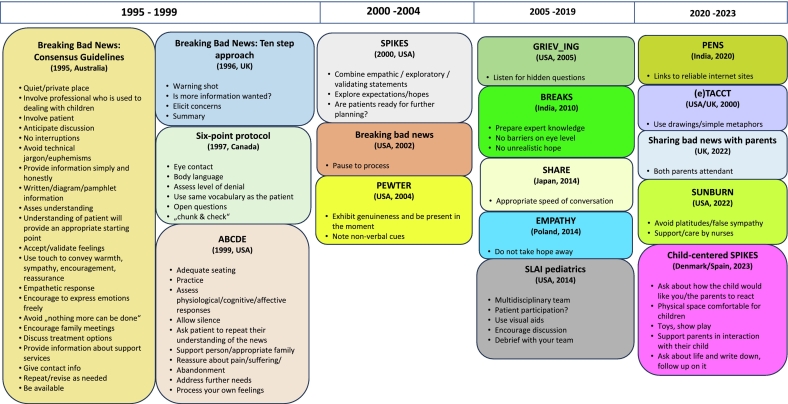
The components extracted from existing BBN protocols that are best suited to the onco-pediatric setting (Map 2).

Ultimately, the segmentation of the 20 BBN protocols (Fig. 3) into their respective components revealed that, despite their disparate design objectives, they exhibit significant component overlap. We identified and extracted those components that applied to PHO. By selecting the ‘initial published components’ from each source and excluding duplicates, 68 unique components were identified from 17 BBN protocols and incorporated into Map 2 (Fig. 4). Most knowledge is based on the Australian consensus guideline [60].

#### 3.2.2 Step 4: new category development

To develop a practical BBN protocol grounded in the research team's experience, the 68 unique components were iteratively organized into 11 categories: specialties that prepare, attendants, find procedure, environment, conversation opening, announcement, reaction, interactive trialogue, information back up, name perspectives, and ongoing support. Then, the categories were allocated to the three BBN phases: preparation (*n* = 4), execution (*n* = 6), and follow-up (*n* = 1). Initially, the findings were developed and presented in a concept map (Map 3). However, as this format proved challenging for individuals unfamiliar with mapping techniques, and in order to augment comprehensibility, the findings were subsequently transferred into a tabular format (Table 1).

#### 3.2.3 Step 5: concept finalization

With this clear structure in place, we condensed the information from the 68 components and develop practical instructions. Given the experience gained by OKRA [37], an appropriate vocabulary was selected for use in pediatric oncology. This approach relied on constructive recommendations rather than categorical prohibitions of specific phrases and expressions. Table 2 presents this operationalization for the German PHO, extending the conceptual framework of Table 1 by translating the theoretical components into concrete communication strategies and implementation examples.

**Table 2:** Development of “SAFE & CARING” from existing protocols: operationalization of theoretical components.

Items from existing protocols	NEW acronym & memory	Proposed new steps
• multidisciplinary team (Wolfe et al., 2014) • support/care by nurses (Velez et al., 2022) • “Involve professional who is used to dealing with children” (Girgis & Sanson-Fisher, 1995)	**S** **Staff that prepare**	• Interdisciplinary team (e.g., MDs, nurses, psychosocial service) • If necessary, involve other stakeholders (e.g., specialized outpatient palliative care)
• Both parents (Kavanagh & Mukhtyar, 2022)/support person/appropriate family (Rabow & McPhee, 2000) • Patient participation (Wolfe et al., 2014)	**A** **Attendants**	• Both parents/all caregivers/reference persons • Discuss participation of the child together
• Anticipate discussion (Girgis & Sanson-Fisher, 1995) and practice (Rabow & McPhee, 2000) • Prepare expert knowledge (Narayanan et al., 2012)	**F** **Familiarization**	• Obtain case-specific expert knowledge • Anticipate conversation and practice concretely
• Quiet/private place (Girgis & Sanson-Fisher, 1995) • Adequate seating (Rabow & McPhee, 2000) • Physical space comfortable for children/toys, show play (Høeg et al., 2023) • No barriers on eye level (Narayanan et al., 2012)	**E** **Environment**	• Quiet/private place • Adequate seating arrangements that enable communication at eye level without barriers • Child-friendly environment (toys, playful environment)
	**&**	
• No interruptions (Girgis & Sanson-Fisher, 1995) • Open questions (Garg et al., 1997) • Ask about life and write down, follow up on it (Hoeg et al., 2023) • Assess understanding (Girgis & Sanson-Fisher, 1995) / level of denial (Garg et al., 1997), response will provide an appropriate starting point (Girgis & Sanson-Fisher, 1995) • Is more information wanted (Kaye et al., 1996)	**C** **Conversation opening**	• Prevent interruptions • Build therapeutic relationship (use method of open questions, to explore everyday life of the pediatric patient and the family) • Ask about level of knowledge and expectations
• Warning shot (Kaye, 1996) • Eye contact (Garg et al., 1997) • Provide information simply and honestly (Girgis & Sanson-Fisher, 1995) • Use same vocabulary (Garg et al., 1997) • Use visual aids (Wolfe et al., 2014), e.g. drawings (Cole & Bird, 2013) • Appropriate speed of conversation (Fujimori et al., 2014) • Avoid technical jargon/euphemisms (Girgis & Sanson-Fisher, 1995) / platitudes/false sympathy (Velez et al., 2022) • Avoid “nothing more can be done” (Girgis & Sanson-Fisher, 1995)	**A** **Announcement**	• Make eye contact and give “warning shot” (some introducing words that make the patient and parents aware) • Transmit the information in an appropriate manner for the target audience (simple, honest, use the same vocabulary as the patient, appropriate speed of conversation) • Use visual aids (e.g. drawings, painting) • Send support signals
• Pause to process (Penson, Slevin, 2002) • Allow silence (Rabow, McPhee, 1999) • Encourage to express feelings freely, accept/validate feelings (Girgis, Samson-Fisher, 1995) • Provide tissues (Girgis, Samson-Fisher, 1995) • “Reassure about pain, suffering, abandonment” (Rabow, McPhee, 1999) • Body language (Garg et al., 1997) • Ask about how the child would like you/the parents to react (Hoeg et al., 2023) • Appropriate touch (Girgis, Samson-Fisher, 1995) • Empathic answer (Girgis, Samson-Fisher, 1995)	**R** **Reaction**	• Make room for a pause and silence and endure the pause/silence • Reflect on your own body language and body language of your conversation partners • Accept/validate/appreciate feelings • Empathetic response to emotions (appropriate touch, reassurance, tissues) • Explore wishes for support of the child and the family and give assurance of support
• “chunk & check” (Garg et al., 1997) • Encourage discussion (Wolfe et al., 2014) • Combine empathetic/exploratory/validating statements (Baile et al., 2000) • Explore patient's expectations/hopes (Baile et al., 2000), no unrealistic hope (Narayanan et al., 2012), do not take away realistic hope (Witt & Jankowska, 2018) • Encourage family meetings (Girgis & Sanson-Fisher, 1995) • Written/diagram/pamphlet information (Girgis & Sanson-Fisher, 1995) / links to reliable internet sites (Mailankody et al., 2020) • Repeat/revise as needed (Girgis & Sanson-Fisher, 1995) • Ask patient to repeat their understanding of news (Rabow & McPhee, 2000) • Support parents in interaction with their child (Høeg et al., 2023)	**I** **Interactive trialogue and information sharing**	• Enable open trialogue (“chunk & check”, repeat information, combine empathetic/exploratory/validating statements, ask patient to repeat what has been understood) • Explore hopes/expectations of the patient, give realistic hopes • Give written/graphic/child appropriate information and reliable sources • Encourage family meetings and give the parents guidance on interacting with their child
• Summarize (Garg et al., 1997) • Discuss treatment options (Girgis & Sanson-Fisher, 1995) • Address further needs (Rabow & McPhee, 2000) • Provide contact information (Girgis & Sanson-Fisher, 1995)	**N** **Name perspectives**	• Provide orientation regarding treatment options • Make an appointment to discuss treatment options • Summarize the conversation • Address open questions • Reassure availability (e.g., phone number, email, …)
• Provide information about support services (Girgis & Sanson-Fisher, 1995) • Debrief with team members (Wolfe et al., 2014) • Process your own feelings (Rabow & McPhee, 2000) • Be available (Girgis & Sanson-Fisher, 1995)	**G** **Give support**	• Team debriefing • Install family specific support (give information about support services, be available) • Initiate coping strategies with team members to process the conversation

#### 3.2.4 Step 6: new protocol creation

Finally, an appropriate name for the protocol was developed through a co-creative process within the team, which would also serve as an acronym for the categories identified. Therefore, synonyms were sought for each category to form words from the initial letters. This creative process was conducted to find an acronym that evokes a positive connotation, is appropriate for use in pediatric oncology, and is easily memorable.

**Fig. 5:**
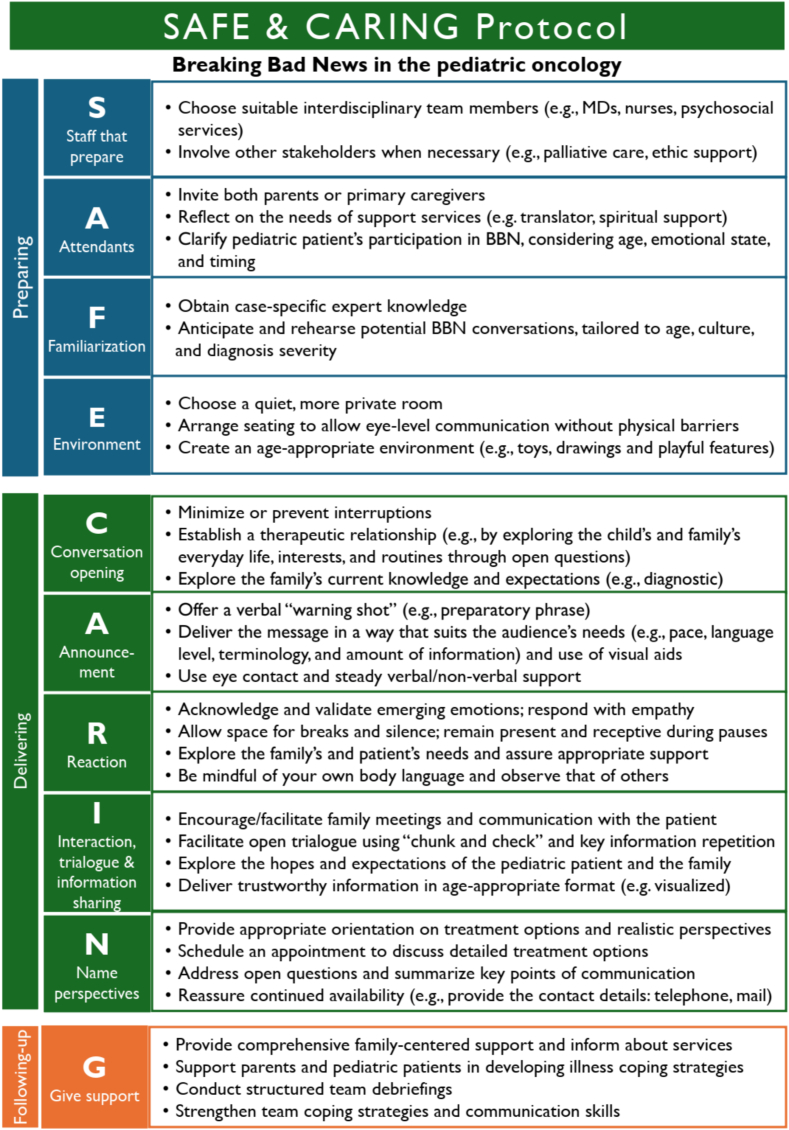
“SAFE & CARING” protocol for BBN in PHO.

The final outcome is the proposed PHO-specific protocol designated “SAFE & CARING,” which consists of ten steps (Fig. 5). “SAFE” focuses on the preparation phase of breaking bad news (BBN): **S**taff preparation, **A**ttendants, **F**amiliarization, and **E**nvironment. The “&” symbol serves as a transitional pause, leading to the “CARING” component, which addresses the transmission and follow-up phases. “CARING” includes: **C**onversation opening, **A**nnouncement, **R**eaction, **I**nteractive trialogue and information back-up, **N**ame perspective, and finally, **G**ive support. A German version of this protocol is provided in Annex 2.

## 4 Discussion and conclusion

### 4.1 Discussion

Resulting from a mapping review, our study (1) provides the first comprehensive overview of existing BBN protocols across healthcare and, in doing so, highlights the urgent need for a PHO-specific approach, and (2) offers a context-sensitive conceptualization tailored to the interprofessional and triadic communication demands involved in the preparation, delivery, and follow-up of BBN in German PHO.

#### 4.1.1 Mapping the landscape of BBN protocols and identifying gaps in pediatric oncology

A comprehensive understanding of existing BBN protocols and their potential applicability in pediatric oncology was achieved through a mapping review (Fig. 1, step 1–3). While mapping reviews have been more commonly applied in fields such as public health, health system development, and environmental research, their use in biomedical sciences remains relatively rare, which remains relatively uncommon in biomedical research [61], [62], [63], [64]. We believe that the integration of visualization into scientific publications provides substantial benefits. It allows complex findings to be communicated more plainly, facilitates rapid identification of trends and gaps, and enhances the accessibility of results for diverse audiences [65]. In our study, we found that visualizing the existing BBN landscape was particularly helpful for establishing a common working ground to develop a new protocol in a co-creative, interdisciplinary manner.

Our mapping review proved particularly obliging for identifying, cataloging, describing, and visualizing evidence across the broad BBN landscape, as well as for highlighting gaps relevant to PHO (Annex 1, Fig. 3, Fig. 4).

##### 4.1.1.1 The historical appearance of BBN protocols

Our results reveal the historical emergence of BBN protocols (Annex 1 and Fig. 3), pinpointing the first protocol in 1995 as a starting point for understanding their evolution in healthcare [60]. Our findings suggest that the foundational period for BBN protocols occurred between 1995 and 2005, during which seven protocols were published. A renewed wave of interest has emerged from 2010 to 2023, which appears to be ongoing, reflecting continued development and attention in the field. The observed increase in publications on BBN protocols likely reflects growing awareness of the importance of structured communication in clinical practice. This trend is consistent with practice-based evidence highlighting challenges in breaking bad news, as well as the integration of communication training into medical curricula, which has emphasized the need for standardized approaches [29], [30], [31], [32], [33].

##### 4.1.1.2 Geographic distribution

Our analysis indicates that the first BBN protocol was developed in Australia using a Delphi process. Among the 28 protocols included in our detailed review, 16 originated from predominantly English-speaking countries, while twelve came from other regions, including Japan, Germany, and Pakistan. This pattern was consistent among the 20 protocols that informed the development of our new PHO protocol, with 14 from English-speaking countries and six from elsewhere, and none from Germany. These findings suggest that BBN protocol development has been concentrated in Anglophone contexts, highlighting a gap in German-language and potentially culturally adapted guidance, which underlines the need for context-specific protocols in settings such as German PHO. The predominance of English-language publications reflects a broader trend in biomedical research, where Anglophone countries dominate protocol development [66]. Moreover, we believe that this is compounded by barriers such as publication fees and language skills, which may limit the visibility of protocols from non-English-speaking regions, including German-speaking countries. As a result, many locally developed BBN protocols likely remain unpublished in English.

##### 4.1.1.3 Scoping BBN protocols across healthcare domains and identifying the gap

Our analysis shows that BBN protocols have predominantly addressed general healthcare settings (14 of 28 protocols), with fewer protocols tailored to oncology (3) and pediatrics (2). Single protocols were identified in specialized domains such as death notification, palliative care, disclosure of medical errors, generic counseling, cultural disclosure, acute and trauma care, neonatology, and oral medicine. Notably, only one protocol published in 2023 specifically targeted pediatric oncology and was based on the SPIKES protocol [19], [55]. While general BBN protocols provide valuable guidance across healthcare, our findings suggest that they may not adequately address the unique communication challenges in specialized settings such as PHO.

The SPIKES protocol provide valuable guidance and is frequently applied in the training of students and HCP for communication of BBN in general [29], [30], [31], [32], [33]. However, our practical experiences in the oncology and also literature indicates several challenges when applying it in the German context [41], and we believe that adaptions on it, like NEO-SPIKES [67] are therefore even less suitable for PHO. Despite broad interest in BBN across healthcare, our findings show that protocols for pediatric oncology are rare, as existing approaches largely build on adaptations of SPIKES and fail to reflect the interdisciplinary, triadic communication reality of PHO.

##### 4.1.1.4 Selecting components of existing BBN protocol for PHO adaptation

In reviewing 20 protocols in detail, we found that only 17 contained elements suitable for adaptation to the PHO context, yielding a total of 68 components (Fig. 4). This process was particularly instructive, as it revealed both the common foundations of BBN—rooted in the original Australian protocol—and the predominant focus of existing approaches on dialogue between adult patients and practitioners. Two decades ago, communication was often regarded as a secondary, ‘nice-to-have’ skill for physicians [68], [69], and the resulting orientations, which dominated BBN practice between 1995 and 2004. It does not sufficiently account for the interdisciplinary, triadic communication processes that characterize PHO.

In 2014, the first BBN protocol addressing pediatrics incorporated the role of multidisciplinary teams and the need for debriefings [55]. More recent protocols have raised questions about pediatric patient participation and introduced family-sensitive elements, such as ensuring both parents attend [70]. Subsequent contributions emphasized language, visual aids, and physical space.

However, none of the protocols were originally developed for PHO and fail to address the complex triadic communication between the interdisciplinary HCP team, parents, and pediatric patient [3], [56]. In line with the MRC framework for complex interventions [71], these findings underscore the need to tailor BBN protocols to the specific healthcare setting, addressing local practices, team structures, and the unique communication challenges of PHO.

#### 4.1.2 Strengths and limitations

This study applied an exploratory mapping review to identify and structure existing BBN protocols—a method suited to heterogeneous, practice-oriented fields—providing conceptual breadth and visual synthesis, though without the exhaustive depth of a systematic review [47], [48].

Despite a broad, iterative search using multiple Boolean strings across databases and citations without date restrictions, some relevant tools using different terminology may have been missed.

To ensure a broad knowledge base, BBN protocols from various disciplines were included, with non-English protocols considered if available in English. Limiting inclusion to published protocols strengthened credibility, though reliance on English/German abstracts and the absence of standardized quality appraisal remains limitations.

Using document analysis and collaborative concept mapping [48], Phase 1 risked bias from a single researcher, while Phase 2 incorporated three German healthcare perspectives to enhance acceptability. Nonetheless, the perspectives of pediatric patients and families were not included due to resource constraints.

The protocol was tailored to the German healthcare system, its generalizability to other contexts remains uncertain.

### 4.2 Innovation

Based on the identified gap, we developed the ‘SAFE & CARING'. To our knowledge, the ‘SAFE & CARING' protocol is the first explicitly designed to address BBN in PHO rather than being adapted from existing protocols. It is intended for German PHO, and its key innovation lie in the collaborative development process, the application of a triadic communication approach, the conceptualization of BBN as a process, and framing BBN as interprofessional responsibility*.* The following sections elaborate on these key innovative features.

#### 4.2.1 Collaborative development of the SAFE & CARING protocol for German PHO

The ‘SAFE & CARING' protocol was developed by systematically merging theoretical evidence with practical experience, reflecting an innovative approach that builds on existing knowledge (Fig. 4). Components from 17 BBN protocols were critically examined and sorted to the BBN phases (Table 1), and finally adapted, resulting in a protocol specifically designed to meet the current needs of German PHO (Fig. 5). In line with the literature, merging theoretical evidence with practical experience strengthens the foundation of protocols and enhances their relevance in real-world settings [72]. After selecting potentially suitable components (by FL), the other three authors contributed contextual knowledge from the OKRA project and extensive clinical experience to develop the protocol [25], [34], [35].

#### 4.2.2 Triadic communication as a core design principle

In PHO, effective triadic communication (child–parent–HCP) requires specific elements that go beyond the traditional dyadic (parent–HCP) dialogue [73]. The central innovation of our protocol is the triadic approach, which has not been explicitly addressed in previous BBN protocols but warrants focused attention. As BBN processes should maintain continuous communication [8], this approach runs like a “common thread” through our protocol, sensitizing healthcare professionals to this aspect. Within `SAFE´ (preparation phase), the letter A – *Attendants* – emphasizes clarifying the patient's and parents' participation, while F – *Familiarization* – and E – *Environment* – highlight considerations essential for conducting a trialogue rather than a dialogue.

In ‘CARING’ (delivery and follow-up), the triadic approach is reflected in each letter, consistently addressing both the pediatric patient and the family. Strategies to facilitate their participation include breaking information into smaller chunks, using plain and direct language, and incorporating visual aids [5], [74]. Attention should also be given to the parents' health literacy and the child's age to prevent emotional and cognitive overload [14].

Meanwhile, developmental considerations, such as age-related attention capacity, may inform how HCPs structure conversations: Younger children (e.g., 2 years) typically sustain attention for only a few minutes, whereas school-aged children (e.g., > 7 years) can engage for longer periods when actively involved. Age-appropriate strategies, such as short, clear statements and opportunities for feedback, may help facilitate meaningful triadic exchange [75].

In general, triadic communication should acknowledge differences in autonomy, cognitive capacity, and emotional processing [14], [56], [73]. For younger or nonverbal children, communication may primarily be directed toward the parents while maintaining visual engagement with the child and using a calm and reassuring tone, resulting in a functionally dyadic interaction despite the child's presence [13], [14], [55], [74]. Following the two-step approach proposed in OKRA which is associated to this study [37], parents may initially receive the diagnosis to allow for initial processing, followed by age-appropriate trialogical communication with the child within a short time frame (see “Recommendation 12” in Annex Overview – Recommendations and Their Categories) [39]. In contrast, when communicating with adolescent patients, HCPs should actively elicit the patient's understanding, questions, and concerns together with those of the parents, thereby engaging in a genuinely triadic exchange [13], [15], [56], [73]. Although cultural and religious background has not been explicitly operationalized in the present protocol, existing literature highlights the importance of cultural factors in pediatric communication and identifies shared decision-making as a distinct, culturally sensitive process [76], [77], [78].

Recent literature has highlighted helpful strategies for BBN [73], [79]. When viewed in this context, our protocol—developed for PHO in Germany—addresses several of these approaches. It sensitizes HCPs to structured preparation, family-specific needs, and supportive environments; emphasizes developmentally appropriate communication across all phases; integrates interprofessional collaboration and role clarification; and highlights active listening and feedback during delivery and follow-up.

#### 4.2.3 Conceptualizing BBN as a structured process

BBN is typically described as a three-phase process: preparation, transmission, and follow-up [9]. Surprisingly, existing protocols rarely outline these phases clearly. Based on our practical experience, protocols are more user-friendly when structured according to this temporal order. The ‘SAFE & CARING' protocol reflects this by encompassing two components: ‘SAFE’ for preparation and ‘CARING' for transmission and follow-up.

We consider the SAFE elements, including careful preparation of the delivery, reflection on who will be involved in the BBN (HCP as well as pediatric patients and their families), familiarization with family-specific needs, and thoughtful preparation of the environment, are essential to ensuring the quality and effectiveness of the BBN. The F – Familiarization – represents an innovative element in our protocol and a key differentiator, as it emphasizes tailoring discussions to a child's age and developmental stage, recognizing that understanding of illness and death varies accordingly. This requires adapting the language, level of detail, and emotional tone of the conversation to the child's needs [80].

Although follow-up has received limited attention in literature and practice, integrating insights from the OKRA project sensitized us to all three BBN phases. In our protocol, the final letter ‘G’ in ‘CARING’ - ‘give support’ - encompasses emotional and structural support for patients, families, and HCPs. We recommend establishing support structures for BBN providers, including debriefing, coping strategies, communication training, and both internal and external supervision within PHO teams.

In order to support HCPs from the outset, we created a memorable acronym that acts as a mental ‘handrail,’ guiding them through emotionally challenging situations and enabling practical, patient- and family-centered care in pediatric oncology.

#### 4.2.4 BBN as an interprofessional responsibility

A persistent challenge in German healthcare is `silo thinking´ where problems are addressed in isolation [81], [82]. Often, BBN is limited to biomedical aspects and delivered exclusively by physicians [68], [69]. In contrast, a systems-thinking perspective acknowledges the complexity of BBN and incorporates input from multiple professionals [81]. The ‘SAFE & CARING' protocol frames BBN as an interdisciplinary responsibility, integrating medical, nursing, and public health perspectives, consistent with evidence demonstrating the benefits of interdisciplinary approaches for patient care and outcomes [83]. In preparation, each discipline contributes to a comprehensive understanding of the family's needs, knowledge, and expectations. During delivery, professionals complement each other—clarifying information, validating emotions, allowing pauses, and addressing the distinct needs of child and parents. In follow-up, this approach reduces information loss while enhancing coping and collective learning, as highlighted by others [42], [55].

### 4.3 Conclusions

#### 4.3.1 Summary of this study

This study provides a comprehensive visual overview of existing BBN protocols across healthcare from 1995 to the present. A critical gap was identified: the absence of a PHO-specific protocol explicitly designed to address triadic communication, particularly in Germany. To address this need, we conducted a collaborative concept-mapping process that integrated theoretical and practice-based evidence, resulting in the development of the context-specific ‘SAFE & CARING' protocol.

‘SAFE & CARING' offers a structured, hands-on first-aid tool for interdisciplinary PHO teams. Its key innovations include the collaborative development process, the integration of triadic communication as an overarching principle, the conceptualization of BBN as a process, and the framing of BBN as an interprofessional responsibility. The protocol may be particularly valuable in PHO settings where formal training in triadic communication is limited. Although developed for the German healthcare context, its underlying principles may inform adaptation in other systems, provided appropriate local validation is undertaken.

#### 4.3.2 Implications for research and practice

In settings without a comprehensive BBN strategy, practical guidance for interdisciplinary PHO teams is urgently needed. BBN protocols can serve as a ‘first aid’ tool, providing structured communication to reduce distress for families and HCPs in complex situations [19], [22], [84], and fostering team collaboration, role clarity, coordination, and efficiency, particularly in follow-up care [85].

Research in adult oncology shows that a comprehensive BBN strategy reduces emotional stress for HCPs [40], [85]. In PHO, context-specific protocols, training and further studies on effective triadic communication strategies across different age groups are needed [1], [22], [56], [73], [86].

The ‘SAFE & CARING' protocol was developed for German PHO, but its impact on care coordination, team emotional strain, and compassionate, consistent communication requires real-world validation. Although developed for Germany, the underlying principles of the protocol may inspire adaptation in other healthcare systems, provided appropriate local validation is conducted.

## Article summary

Through a mapping review, we provide a comprehensive overview of current BBN protocols, resulting in the creation of a new protocol for pediatric oncology.

## What's known on this subject

BBN is considered challenging in pediatric oncology. Besides profound medical knowledge, triadic communication skills and a multi-perspective understanding of patients' needs are required. There is a lack of overview of existing BBN protocols and context-specific guidelines, with focus on triadic communication.

## What this study adds

BBN evidence was scoped and mapped to provide a chronological, geographical, and substantive overview. Maps were used to initiate a co-creative process that integrated theoretical and practical evidence to create the onco-pediatric specific BBN protocol “SAFE & CARING.”

## CRediT authorship contribution statement

**Theresia Krieger:** Writing – original draft, Validation, Methodology, Investigation, Formal analysis, Data curation, Conceptualization. **Lisa Frey:** Writing – original draft, Investigation, Formal analysis, Data curation, Conceptualization. **Marc Hoemberg:** Writing – review & editing, Validation, Resources. **Kerstin Dittmer:** Writing – review & editing, Visualization, Validation, Supervision, Resources.

## Funding

No funding was received for this study.

## Declaration of competing interest

We, the authors declare that they have no known competing financial interests or personal relationships that could have appeared to influence the work reported in this paper.

## Figures and Tables

**Table:** 

Nr	Acronym/name/year	Author/publication	Design intention
1	Breaking Bad News: Consensus Guidelines [1995]	Girgis A, Sanson-Fisher RW. Breaking bad news: consensus guidelines for medical practitioners. J Clin Oncol. 1995 Sep;13(9):2449–56. doi:https://doi.org/10.1200/JCO.1995.13.9.2449	BBN in general
2	Breaking Bad News: A ten step protocol [1996]	Kaye, P. *Breaking Bad News: A ten step approach*. 1996. Scion Publishing Ltd., 2023.	BBN in general
3	Six-Point Protocol [1997]	Garg A, Buckman R, Kason Y. Teaching medical students how to break bad news. CMAJ. 1997 Apr 15;156(8):1159–64. PMID: 9141988; PMCID: PMC1227243.	BBN in general
4	ABCDE [2000]	Rabow MW, McPhee SJ. Beyond breaking bad news: Helping patients who suffer. *BMJ*. 2000;320(Suppl S3):365. doi:https://doi.org/10.1136/sbmj.000365	BBN in general
5	SPIKES [2000]	Baile WF, Buckman R, Lenzi R, Glober G, Beale EA, Kudelka AP. SPIKES-A six-step protocol for delivering bad news: application to the patient with cancer. *Oncologist*. 2000;5(4):302–311. doi:https://doi.org/10.1634/theoncologist.5-4-302	BBN in oncology
6	Breaking Bad News [2002]	Penson, R. T., & Slevin, M. L. (2002). Communication with the cancer patient. In *Treatment of Cancer* (Fourth edition, S. 1187–1198). Arnold.	BBN in oncology
7	GRIEV_ING [2005]	Hobgood, C. (2005). Delivering the news with compassion: The GRIEV_ING death notification protocol. Online verfügbar unter https://www.acep.org/siteassets/uploads/uploadedfiles/acep/clinical-and-practicemanagement/resources/academics/deathnotification, zuletzt geprüft am 25.06.2024.	Death notification protocol
8	SAGE & THYME [2010]	Connolly, M., Perryman, J., McKenna, Y., Orford, J., Thomson, L., Shuttleworth, J., & Cocksedge. (2010). SAGE & THYME™: A model for training health and social care professionals in patient-focussed support, Patient Education and Counseling. *Elsevier Irland Ltd.*, *79*(1), 87–93. https://doi.org/10.1016/j.pec.2009.06.004	Communication skills in end-stage respiratory disease
9	BREAKS [2010]	Narayanan V, Bista B, Koshy C. ‘BREAKS’ Protocol for Breaking Bad News. *Indian J Palliat Care*. 2010;16(2):61–65. doi:https://doi.org/10.4103/0973-1075.68401	BBN in general
10	(e)TACCT [2014]	Cole, S. A., & Bird, J. (2014). Breaking bad news. In *The Medical Interview E-Book: The Three Function Approach* (third edition, p. 216–221). ELSEVIER SAUNDERS.	BBN in general
11	SLAI pediatrics [2014]	Wolfe AD, Frierdich SA, Wish J, Kilgore-Carlin J, Plotkin JA, Hoover-Regan M. Sharing life-altering information: development of pediatric hospital guidelines and team training. *J Palliat Med*. 2014;17(9):1011–1018. doi:https://doi.org/10.1089/jpm.2013.0620	BBN in pediatrics
12	SHARE [2014]	Fujimori M, Shirai Y, Asai M, Kubota K, Katsumata N, Uchitomi Y. Effect of communication skills training program for oncologists based on patient preferences for communication when receiving bad news: a randomized controlled trial. *J Clin Oncol*. 2014;32(20):2166–2172. doi:https://doi.org/10.1200/JCO.2013.51.2756	BBN in oncology
13	CONES [2014]	Fujimori M, Shirai Y, Asai M, Kubota K, Katsumata N, Uchitomi Y. Effect of communication skills training program for oncologists based on patient preferences for communication when receiving bad news: a randomized controlled trial. *J Clin Oncol*. 2014;32(20):2166–2172. doi:https://doi.org/10.1200/JCO.2013.51.2756	Disclosing mistakes in the medical context
14	DISHA [2015]	Kashewar, S. G., & Kashewar, S. S. (2015). *Training of medical interns in breaking bad news using DISHA protocols*. *3*(4), 161–164. https://doi.org/10.5455/jcme.20151117114244	BBN in general
15	PACIENTE [2017]	Pereira, C. R., Calônego, M. A. M., Lemonica, L., & Barros, G. A. M. de (2017). The P-A-C-I-E-N-T-E Protocol: An instrument for breaking bad news adapted to the Brazilian medical reality. *Revista Da Associação Médica Brasileira*, *63*(1), 43–49. https://doi.org/10.1590/1806-9282.63.01.43	BBN in general
16	Truth telling protocol [2017]	Abazari, P., Taleghani, F., Hematti, S., Malekian, A., Mokarian, F., Hakimian, S. M. R., & Ehsani, M. (2017). Breaking bad news protocol for cancer disclosure: an Iranian version. *Journal of medical ethics and history of medicine*, *10*, 13	BBN in oncology in Iran
17	PEWTER [2018]	Keefe-Cooperman K, Savitsky D, Koshel W, Bhat V, Cooperman J. The PEWTER Study: Breaking Bad News Communication Skills Training for Counseling Programs. *Int J Adv Counseling*. 2018;40(1):72–87. doi:https://doi.org/10.1007/s10447-017-9313-z	BBN in general
18	Breaking bad news: a contextual model for Pakistan [2018]	Baig, L., Tanzil, S., Ali, S. K., Shaikh, S., Jamali, S., & Khan, M. (2018). Breaking Bad News: A contextual model for Pakistan. *Pakistan journal of medical sciences*, *34*(6), 1336–1340. https://doi.org/10.12669/pjms.346.15663	BBN in general, how to avoid violence while BBN
19	EMPATHY [2018]	Witt, M.M.; Jankowska, K.A. (2018): Breaking bad news in genetic counseling—problems and communication tools. *J Appl Genetics* 59, S. 449–452. https://doi.org/10.1007/s13353-018-0469-y	BBN in genetic counseling
20	MR. SPIKES [2019]	Cannone, Daniel; Atlas, Mark; Fornari, Alice; Barilla-LaBarca, Maria-Louise; Hoffman, Mark (2019). Delivering Challenging News: An Illness-Trajectory Communication Curriculum for Multispecialty Oncology Residents and Fellows. *MedEdPORTAL: the journal of teaching and learning resources* 15, S. 10819. DOI: 10.15766/mep_2374-8265.10819	BBN in oncology
21	PENS [2021]	Mailankody, S., Rao, S.R. “PENS” approach for breaking bad news—a short and sweet way!. *Support Care Cancer* 29, 1157–1159 (2021). https://doi.org/10.1007/s00520-020-05807-0	BBN in oncology
22	ARCHES [2021]	Holmes SN, Illing J. Breaking bad news: tackling cultural dilemmas. *BMJ Supportive & Palliative Care* 2021;11:128–132	BBN when the family has requested diagnostic non-disclosure
23	SUNBURN [2022]	Velez, D.; Gerberding, A; Ahmeti, M. (2022): - SUNBURN: a protocol for delivering bad news in trauma and acute care surgery. In: *Trauma Surgery & Acute Care Open*;7:e000851	BBN in acute care and trauma
24	Sharing bad news with parents [2022]	2022 Kavanagh, C; Mukhtyar B. (2022). Parents or carers of children with newly diagnosed neurological; impairment or disability or other severe chronic or life threatening illness. Online available. /Sharing-Bad-News-with-Parents-JCG0325v3.1–2.pdf	BBN to parents
25	CONNECT [2022]	Sobczak K. (2022): The “CONNECT” Protocol: Delivering Bad News by Phone or Video Call. In: *Int J Gen Med*. 2022;15:3567–3572, https://doi.org/10.2147/IJGM.S358723	BBN in general by phone or video call
26	NEO-SPEAK [2022]	Seifart, Carola; Falch, Mirjam; Wege, Mirjam; Maier, Rolf F.; Pedrosa Carrasco, Anna J. (2022): NEO-SPEAK: A conceptual framework that underpins breaking bad news in neonatology. *Front. Pediatr.* 10, Artikel 1,044,210, S. 1044210. DOI: https://doi.org/10.3389/fped.2022.1044210.	Framework for BBN in neonatology
27	Protocol for breaking bad news [2023]	Alves, C.G.B., Santos-Silva, A.R., Martins, B.N.F.L., Scarini, J.F. (2023). Protocol for Breaking Bad News. In: Santos-Silva, A.R., Lopes, M.A., Scarini, J.F., Vargas, P.A., Almeida, O.P.d. (eds) Clinical Decision-Making in Oral Medicine. Springer, Cham. https://doi.org/10.1007/978-3-031-14945-0_5	BBN in oral medicine
28	SPIKES, adapted [2023]	Høeg BL, Sevillano PB, Enesco I, Wakefield CE, Larsen HB, Bidstrup PE. Child-centered communication interventions in pediatric oncology: A scoping review and proposed new communication model. *Pediatr Blood Cancer*. 2023:e30533. doi:https://doi.org/10.1002/pbc.30533	BBN in pediatric oncology

**Figure:**